# Can vessel dimension explain tolerance toward fungal vascular wilt diseases in woody plants? Lessons from Dutch elm disease and esca disease in grapevine

**DOI:** 10.3389/fpls.2014.00253

**Published:** 2014-06-12

**Authors:** Jérôme Pouzoulet, Alexandria L. Pivovaroff, Louis S. Santiago, Philippe E. Rolshausen

**Affiliations:** Department of Botany and Plant Sciences, University of CaliforniaRiverside, CA, USA

**Keywords:** Vascular wilt, xylem morphology, compartmentalization, grapevine trunk diseases, esca, *Phaeomoniella chlamydospora*

## Abstract

This review illuminates key findings in our understanding of grapevine xylem resistance to fungal vascular wilt diseases. Grapevine (*Vitis* spp.) vascular diseases such as esca, botryosphaeria dieback, and eutypa dieback, are caused by a set of taxonomically unrelated ascomycete fungi. Fungal colonization of the vascular system leads to a decline of the plant host because of a loss of the xylem function and subsequent decrease in hydraulic conductivity. Fungal vascular pathogens use different colonization strategies to invade and kill their host. *Vitis vinifera* cultivars display different levels of tolerance toward vascular diseases caused by fungi, but the plant defense mechanisms underlying those observations have not been completely elucidated. In this review, we establish a parallel between two vascular diseases, grapevine esca disease and Dutch elm disease, and argue that the former should be viewed as a vascular wilt disease. Plant genotypes exhibit differences in xylem morphology and resistance to fungal pathogens causing vascular wilt diseases. We provide evidence that the susceptibility of three commercial *V. vinifera* cultivars to esca disease is correlated to large vessel diameter. Additionally, we explore how xylem morphological traits related to water transport are influenced by abiotic factors, and how these might impact host tolerance of vascular wilt fungi. Finally, we explore the utility of this concept for predicting which *V. vinifera* cultivars are most vulnerable of fungal vascular wilt diseases and propose new strategies for disease management.

## INTRODUCTION

In vascular plants, xylem functions to conduct water from roots to leaves and provides mechanical support. Yet, xylem is subjected to various biotic and abiotic stresses that threaten its function. The loss of capacity for water transport can come about through loss of xylem vessel function by cavitation in a response to drought or freezing, or by occlusion of vessels by tyloses and gels in response to sapwood-dwelling pathogens. This review examines key findings in our understanding of xylem structure and function in perennial plants and how this relates to resistance of biotic stress from vascular wilt diseases caused by fungi.

Fungal vascular pathogens are capable of utilizing wood polymers as energy sources and are the cause of many economically important diseases in forest trees, ornamental, and agricultural woody crops (Agrios, 2005). The classification schemes of these fungi are based on several features including fungal colonization strategies, macroscopic, and microscopic patterns of wood degradation and ability to degrade certain cell wall polymers (Blanchette, 1995; Pearce, 1996). For instance, vascular wilts and cankers are two separate types of wood diseases. Vascular wilt pathogens cause diseases that lead to wilting of leaves followed by sudden collapse of limbs or entire trees. These symptoms can be due to systemic spread of fungal spores and phytotoxins, as well as the disruption of water flow due to embolism or occlusion of vessels in response to infection (Pearce, 1996; Yadeta and Thomma, 2013). The wilt-causing fungi are mainly restricted to the vessel lumen and cells surrounding vessels until they kill their host (Agrios, 2005). In contrast, fungi causing other vascular diseases such as cankers, usually lead to a slow decline of the plant host, mainly because they do not colonize systemically. Fungi invade bark tissues and xylem, resulting in the death of a portion of vascular cambium (Pearce, 1996). Infected portions of the perennial structure are no longer able to produce newly functional xylem and phloem, and cankers develop. Some vascular fungi have been considered latent opportunistic pathogens and cause diseases such as canker or cane blight, when their host is subjected to abiotic stresses, such as drought (Pearce, 1996; Slippers and Wingfield, 2007; Jactel et al., 2012).

**FIGURE 1:**
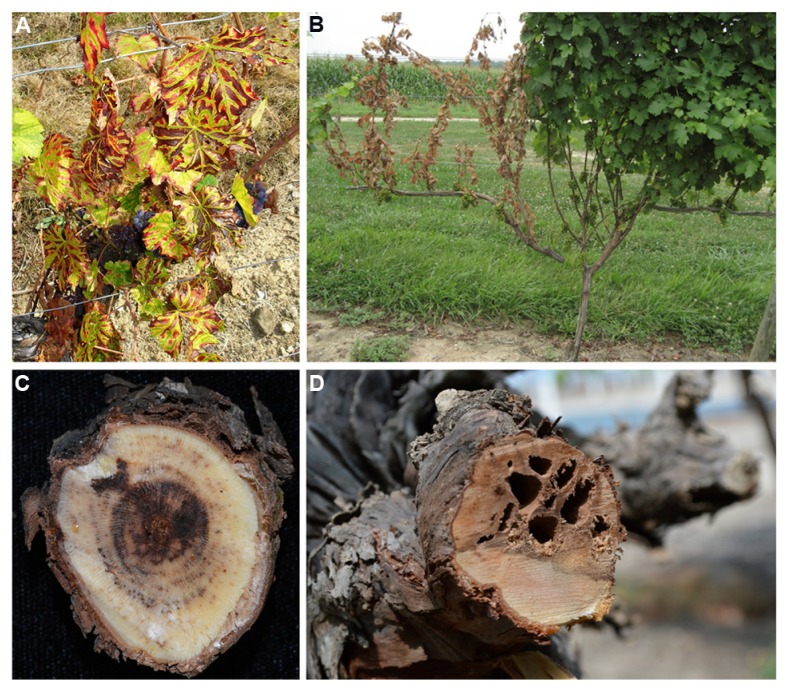
**Symptoms expressed in grapevine affected with fungal vascular diseases. (A)** Vines showing tiger-stripe leaf symptoms associated with the chronic form of esca. **(B)** Symptoms of apoplexy on a young grapevine; note the foliar symptoms between the apparently healthy (right) and apoplectic (left) cordon. **(C)** Cross section of a grapevine wood spur infected with esca; note the necrotic spots in vessels organized in rings and the brown necrosis in the center of the wood. **(D)** Cross section of a grapevine cordon infected with eutypa dieback; note the wedge-shaped canker.

In commercial grapevine (*Vitis vinifera* L.), vascular diseases (e.g., eutypa dieback, botryosphaeria dieback, and esca; see **Figure 1**) are major factors limiting crop productivity. The causal agents are a set of taxonomically unrelated fungi, among which *Eutypa lata, Phaeoacremonium aleophilum, Phaeomoniella chlamydospora, Diplodia seriata*, and *Neofusicoccum parvum* are some of the most virulent and widespread (Gubler et al., 2010; Urbez-Torres, 2011; Travadon et al., 2012; Bertsch et al., 2013). These vascular diseases are detrimental to all viticulture areas worldwide, because they reduce vineyard longevity, cumulative yield, and fruit quality (Munkvold et al., 1994; Mugnai et al., 1999; Lorrain et al., 2012; Bertsch et al., 2013). The point of entry of these pathogenesis primarily through pruning wounds (Rolshausen et al., 2010). Because pruning is a necessary practice to maintain crop yield and quality, fungal vascular diseases are a chronic problem. In addition, there are evidences that plants can become infected in nurseries during the propagation phase (Gramaje and Armengol, 2011), or in vineyards after planting in infested soils (Agustí-Brisach et al., 2013). These alternative infection routes have been clearly demonstrated with *P. chlamydospora,* providing evidence that this pathogen is also soil-borne. While no grapevine is known to be completely resistant to vascular diseases (Bertsch et al., 2013), there are degrees of susceptibility ranging from highly susceptible to tolerant (Péros and Berger, 1994; Feliciano et al., 2004; Christen et al., 2007; Bruez et al., 2013; Travadon et al., 2013; Murolo and Romanazzi, 2014). Studies that have focused on identifying the virulence factors produced by fungi and deciphering the mechanism of pathogenesis have provided clues regarding types of disease and colonization strategy. The recent sequencing of fungal genomes (Blanco-Ulate et al., 2013a,b,c) will also provide insightful information of the molecular mechanisms of pathogenesis and help with the understanding of the disease etiology.

In this “theory and opinion” article, we provide evidence that the wood anatomy and, specifically, xylem vessel diameters differ among grapevine cultivars and that these features could predict the degree of susceptibility to vascular diseases such as those caused by *P. chlamydospora*. To support our hypothesis, we draw a comparison between Dutch elm disease (DED), a well studied wilt disease, and grapevine esca disease. Finally, we explore how vessel dimensions are affected by abiotic factors, and how this concept may be used in agriculture to mitigate economic impacts of vascular wilt diseases.

## XYLEM MORPHOLOGY AND ITS TOLERANCE/SUSCEPTIBILITY TO VASCULAR PATHOGENS

The living sapwood of trees is known to deploy anatomical, physiological, and biochemical features in order to compartmentalize fungal vascular invasion. These responses include the production of resins, pathogenicity related proteins (PRPs), phytoalexins within the cell lumen, as well as plant cell wall thickening (Pearce, 1996). The CODIT (Compartmentalization Of Decay In Trees) and reaction zone models were originally proposed to explain the mechanism of compartmentalization taking place following wounding and infection by decaying fungal pathogens in trees (Shigo and Marx, 1977). The principle of the CODIT lies in the existence of four types of “Walls” aimed at restricting pathogen spread. Wall 1 restricts pathogen movement longitudinally, and is basically associated with vessel occlusions through tylosis and gels. Wall 2 consists of the growth ring boundary and restricts pathogen movement centripetally. Wall 3 limits the tangential movement of pathogen and is associated with ray parenchyma. These first three walls occur in lignified tissues pre-existing injury and can be interpreted as the reaction zone. In contrast, Wall 4, referred to as the barrier zone, is edified by modified cells newly formed after injury and provides a more impervious obstacle toward pathogen spread compared to the three other walls (Pearce, 1996). This wall is critical in infected trees because it maintains newly formed conductive xylem and vascular cambium integrity, thereby promoting plant longevity.

**FIGURE 2:**
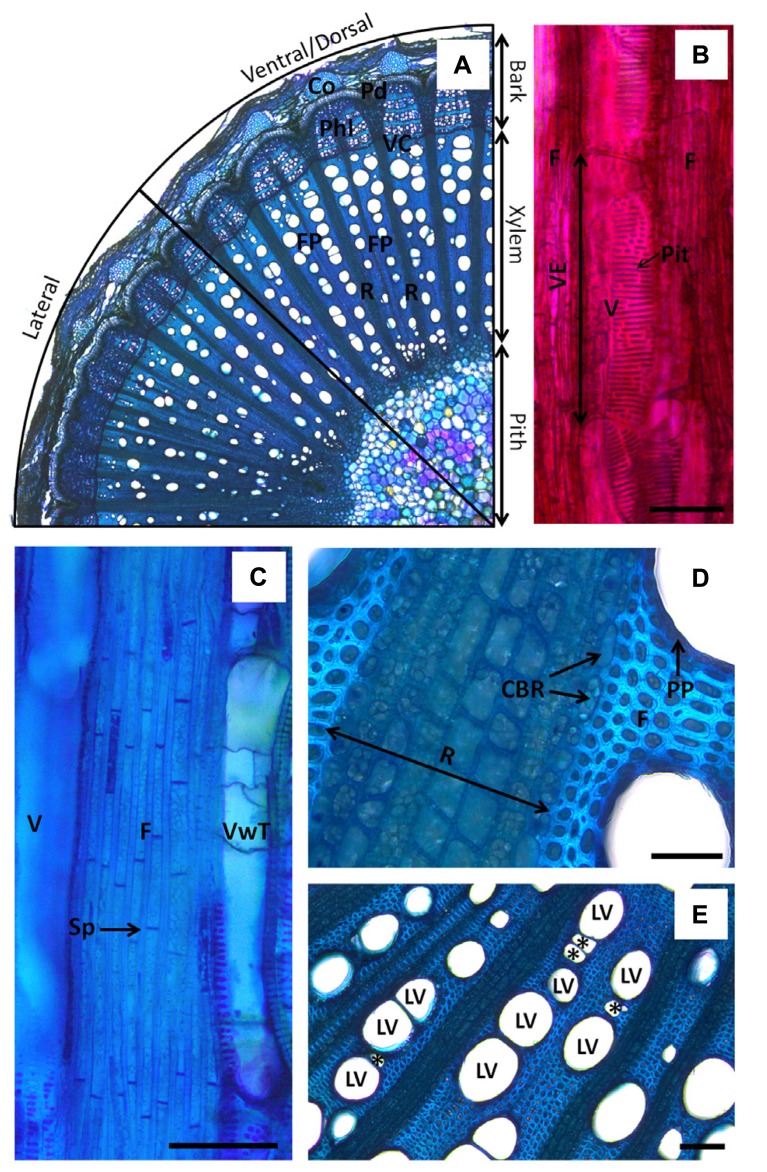
**Anatomy of *Vitis vinifera* xylem. (A)** Micrograph showing organization of stem tissues in cross-section (toluidine blue O). Note the segmentation of xylem in fascicular portions (FP) where large vessels (LV) are packed. Fascicular portions are separated by large rays (R). Note the change in vessel diameters in the lateral and ventral/dorsal sector of the stem. **(B)** Micrograph showing a large vessel in longitudinal section (safranin O). Vessel element (VE) is shown. Note the great area occupied by scalariform pits in the vessel cell wall. **(C)** Micrograph showing septate fibers in longitudinal section (toluidine blue O). **(D)** Micrograph showing close view of ray parenchyma in stem cross-section (toluidine blue O). Note the shape of cells bordering the ray (CBR) compared to ray cells and fibers. Change in staining of the wall due to differential lignification is also observed according to the side in contact with ray cells or in contact with fibers. Note the presence of a single-seriate layer of flat cells forming the paratracheal parenchyma around vessels. **(E)** Micrograph of stem cross-section showing LV in contact with each other or connected by vessel relays (*). Note the presence of LV in contact with ray parenchyma. The notations CBR stands for cells bordering ray, Co is for cork, F is for fibers, FP is for fascicular portion, LV is for large vessel, Pd is for periderm, Phl is for phloem, PP is for paratracheal parenchyma, R is for ray parenchyma, Sp is for septation, V is for vessel, VC is for vascular cambium, VwT is for vessel with tyloses. Scale bars = 100 μm.

In the compartmentalization model, wood anatomy, speed of the host response to infection, and the chemical nature and the spatial organization of wall boundaries in xylem parenchyma, are key elements to explain how compartmentalization of pathogens succeeds or fail amongst and within plant species (Bonsen et al., 1985; Pearce, 1996; Bucciarelli et al., 1999; Deflorio et al., 2009). However, most studies have described the CODIT model in forest and ornamental trees. In grapevine, information on xylem structural modification in response to wounding and pathogen infection is still fragmented. Grapevines are woody vines, or “lianas,” and thus their wood anatomy differs from trees. As a consequence, the wood response to injuries at the structural level presents specific features that differ slightly from that displayed in trees (Pouzoulet et al., 2013). Grapevine stems are bilaterally symmetric structures with distinctive lateral, dorsal and ventral xylem sectors (**Figure 2A**). Dorsal–ventral and lateral sectors can be easily identified in longitudinal sections due to stem shape and the narrower vessel diameters found in lateral sectors, whereas dorsal and ventral sectors are quite similar. Dorsal and ventral sectors of xylem are in fact related to adaxial and abaxial parts of the stem, respectively. Grapevine xylem presents fascicular portions (FP) where large conduits are packed. These FP are separated by large pluri-seriated rays (**Figure 2A**). Conjunctival tissue in FP is only composed of septate living fibers (**Figure 2C**), and uni-seriate paratracheal parenchyma is presented around vessels (Schoch et al., 2004; **Figure 2D**). Solitary large vessels (LV) can be connected by relays composed of a series of narrower vessels or can be connected together directly (**Figure 2E**; Brodersen et al., 2013a). However, some FP can be directly connected by fibers and LV in mature wood where rays seem to spread out in a lesser extent. Rays are surrounded by a one-cell layer that differs slightly from ray cells in shape (Schoch et al., 2004; **Figure 2D**).

**FIGURE 3:**
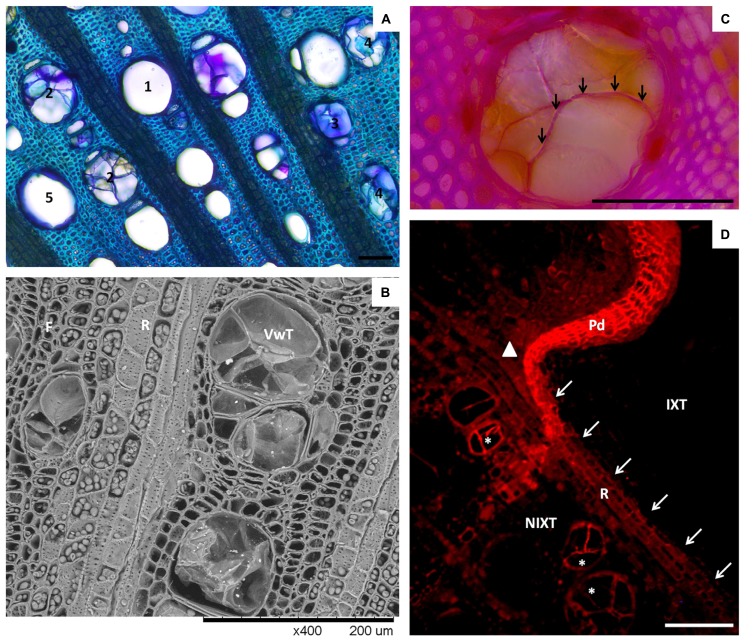
**Vessel occlusions in *Vitis vinifera* xylem. (A)** Micrograph showing different types of occlusion in cross-section of grapevine xylem (bright field, toluidine O). (1) stands for open vessel (no occlusion), (2) stands for vessels occluded by tyloses, (3) stands for vessels occluded by gels, (4) stands for vessels occluded by both tyloses and gels, (5) stands for open vessels harboring a layer of gels coating their walls. **(B)** SEM micrograph of a stem cross-section showing tyloses in LV. Note the abundance of intercellular junctions occurring in the wall of ray parenchyma cells (R). **(C)** Close view of an occluded vessel showing the occurrence of lignin in a tylosis wall (arrows), demonstrated by the deep purple color developed in reaction to phloroglucinol/HCl staining. **(D)** Epifluorescent micrograph showing suberin location in a stem cross-section 2 months after mechanical injury (sudan IV, Ex 590–650 nm/Em > 667 nm). Note the accumulation of suberin in ray (arrows) separating injured (IXT) and non-injured (NIXT) xylem tissues, in the continuity of the periderm (Pd) formed in response to injury. Also, note the presence of a clear signal from the wall of tylosis in some vessels (*). The position of the vascular cambium at the time of injury is indicated by a solid triangle. Scale bars = 100 μm.

A few studies have looked at the grapevine sapwood structural modifications in response to wounding and pathogen infection. In wounded cuttings, characterization of cell wall modifications in living fibers and ray parenchyma around the wound appears to be associated with suberin rather than lignin deposits (Pouzoulet et al., 2013; also see **Figure 3D**). Impregnation of cell walls by phenolic compounds not related to lignin within the reaction zone might also be hypothesized as part of the response to injury (Troccoli et al., 2001; Mutawila et al., 2011). In infected plants, the chemical composition of xylem cell walls might be considered as a factor of tolerance toward fungal vascular pathogens causing canker diseases (Blanchette, 1995). *E. lata* the causal agent of eutypa dieback, causes wedge shaped cankers in grapevine (**Figure 1D**). This fungus is capable of producing an array of cell wall degrading enzymes and phytotoxic secondary metabolites to break down secondary plant cell walls (Elghazali et al., 1992; Rolshausen et al., 2008). Rudelle et al. (2005) looked at the structural modification of infected grapevine xylem tissues and found cell wall thickening in paratracheal parenchyma to impede lateral hyphae penetration in xylem parenchyma cells. In addition, Rolshausen et al. (2008) showed that higher levels of phenolic compounds were measured in wood of resistant cultivar to eutypa dieback, suggesting that constitutive chemical composition of wood plays a role in the variation of effectiveness of pathogen compartmentalization between tolerant and susceptible cultivars. In contrast to *E. lata*, *P. chlamydospora* lacks the ability to produce substantial amounts of enzymes to degrade secondary cell walls (Valtaud et al., 2009). However, like many other vascular wilt fungi (Klosterman et al., 2011), *P. chlamydospora* exhibit pectinolytic activity (Marchi et al., 2001), suggesting that the fungus is able to degrade pectin rich pit membranes connecting xylem vessels, as well as gels secreted in vessels by the host in response to infection. Histopathological studies confirm that *P. chlamydospora* mainly resides in vessels (Valtaud et al., 2009; Fleurat-Lessard et al., 2010), but is also able to progress in vessels occluded by tyloses and gels (Fleurat-Lessard et al., 2010; Mutawila et al., 2011; Pouzoulet et al., 2013). Observations indicate that this pathogen is also able to colonize xylem parenchyma cells, although this is accomplished to a lesser extent (Valtaud et al., 2009; Pouzoulet et al., 2013). Because, *P. chlamydospora* has not been found to be able to alter secondary cell wall of its host, it could benefit from already existing openings in cell walls to spread out from a parenchyma cell. Pouzoulet et al. (2013) observed that suberized layers that developed in ray parenchyma (see **Figure 3D**) could efficiently restrict the fungus spread from one fascicular portion to another. Finally, *P. chlamydospora* is also known to produce many phytotoxins that are translocated with the evapotranspiration stream of the plant (Bruno and Sparapano, 2007; Bruno et al., 2007; Andolfi et al., 2009; Fleurat-Lessard et al., 2010; Luini et al., 2010). Overall, these pathogenic traits match with those observed for other known vascular wilt fungi (Agrios, 2005; Yadeta and Thomma, 2013).

Wood infected with esca disease appears as dark necrotic spots in cross-sections with development of pink/red brown necrosis surrounding the black spots (**Figure 1C**) and black streaking in longitudinal wood sections (Mugnai et al., 1999). Characteristic tiger-stripe leaf symptoms are expressed in infected grapevines (a.k.a., chronic esca) toward the end of the growing season (**Figure 1A**). In some cases, vine apoplexy, or wilt, develops suddenly in the summer, which consists of a sudden collapse of half or the entire affected grapevine (**Figure 1B**). *P. chlamydospora* is also known as the causal agent of the Petri disease, a wilt disease observed in young vineyards in semi-arid areas (Mugnai et al., 1999). Lambert et al. (2013) showed a faster and earlier induction of defense-related genes with higher accumulation of stilbene compounds and pathogenesis-related proteins in grape cultivars resistant to esca dieback. However, no studies have looked at differences in xylem morphology or in structural modification of xylem cells at the site of the infection that could explain the differences insusceptibility observed in *V. vinifera* cultivars.

Vascular wilt pathogens are mainly restricted to vessels, and these agents are able to quickly spread systemically by the means of spores in open vessels. In this context, plant response through vessel occlusion appears to be the most important component for an efficient restriction of infections of these pathogens. In grapevine, amongst the four different walls of the CODIT model described above, vessel occlusion (i.e., Wall 1) has been studied most extensively. Occlusion of vessels in grapevine can be assured by the development of tyloses and gels originating from cells associated with vessels (referred in this article as paratracheal parenchyma; Sun et al., 2008; **Figures 3A,B**). Histological studies regarding the detailed organization and chemical nature of tyloses have been addressed in tree species but not yet in grapevine. Tylosis walls are heterogenic structures composed of distinctive layers (Rioux et al., 1995). The presence and the spatial organization of these layers can vary slightly according to their maturation stages and the species studied (Rioux et al., 1995). Basically, the organization and the chemical nature of the wall of mature tylosis appear to share many similarities with the wall of xylem parenchyma cells. The outer layer seems to be mainly composed of pectin, and can be assimilated in its function to the middle lamella. Inward, a cellulosic primary cell wall, within which pectin can be detected, is present. The edification of a secondary wall can also be observed in some species (Rioux et al., 1995). Primary and secondary tylosis walls can also be reinforced by lignins to various degrees. Finally, tylosis harbor internal suberized layers in which phenolic compounds can accumulate. Multiple internal suberized layers that alternate with internal cellulosic layers were also described in *Populus basalmifera* (Rioux et al., 1995). Pouzoulet et al. (2013) recently reported that ligno-suberized tyloses also develop in grapevine (also see **Figures 3C** and **2D**). Thus, mature tyloses in vessels represent a succession of rigid ligno-suberized barriers that might impede the spread of pathogens. However, it appears that within occluded vessels, narrow spaces remain between mature tyloses, as well as between mature tyloses and the vessel wall (Rioux et al., 1998). Observations indicate that these narrow spaces are filled by anamorphous compounds rich in pectin (gels), secreted outside the tyloses in the vessel’s lumen.

In grapevine, it is still not clear whether tyloses or gels develop preferentially in response to fungal infection. One can assume that the host response mechanism is somewhat driven by the colonization strategy of the pathogens. Mutawila et al. (2011) reported the development of both tyloses and gels in *P. chlamydospora*-infected vessels, and that both structures could be present in the same vessels (also see **Figure 3A**). The development of “black-goo” (**Figure 1C**), typically observed in vessels in response to *P. chlamydospora* infection, seems associated with secretion of gels or gums by the host (Mutawila et al., 2011). In addition, infection of grapevine with *Xylella fastidiosia,* a xylem-dwelling bacterium and causal agent of Pierce’s disease, induced both vessel occlusion by tyloses and gels, but with a large preference for tylosis occlusion (Sun et al., 2013). However, other factors may influence the nature of the host response as well. For instance, Sun et al. (2008) observed that in response to wounds, tyloses formation occurs preferentially in the summer, while gels preferentially form in the winter. Sun et al. (2007) also showed that occlusion of vessels occurs in response to hormonal signals (i.e., ethylene) known to be involved in plant response to wounding, but also in response to infection.

## VESSEL SIZE AND TOLERANCE TO FUNGAL VASCULAR WILT DISEASES IN ELM AND GRAPEVINE

In this article, we establish a parallel between DED and grapevine esca disease in order to explain differences in susceptibility observed amongst plant genotypes. DED is a well studied vascular wilt disease caused by the ascomycete fungi *Ophiostoma ulmi* and *O. novo-ulmi* (Bonsen et al., 1985). In this pathosystem, wilting is assumed to be caused by the development of vessel embolisms consecutive to fungal infection, rather than the spread of toxins (Newbanks et al., 1983). Recent findings demonstrate that plant genotypes with high susceptibility to DED were found to have a greater number of wide diameter vessels than those with low susceptibility (Solla and Gil, 2002a,b; Martín et al., 2009; Venturas et al., 2013). Solla and Gil (2002b) proposed that in DED, xylem cavitation is first induced in the vessels of greatest diameter while small vessels are less affected by DED and they continue to conduct sap, as it has been observed in the case of drought stress induced embolisms (Hargrave et al., 1994). However, the difference in vessel morphology was recently found to be unrelated with xylem tolerance to drought in the elm genotype studied (Venturas et al., 2013). These findings suggest that in DED, vessel dimension plays a role in the ability of the tree to compartmentalize the pathogen. As we will discuss further in this chapter, greater blocking of vessels, which contributes to compartmentalizing the disease (Solla and Gil, 2002b), could also occur in vessels of lower diameter.

**FIGURE 4:**
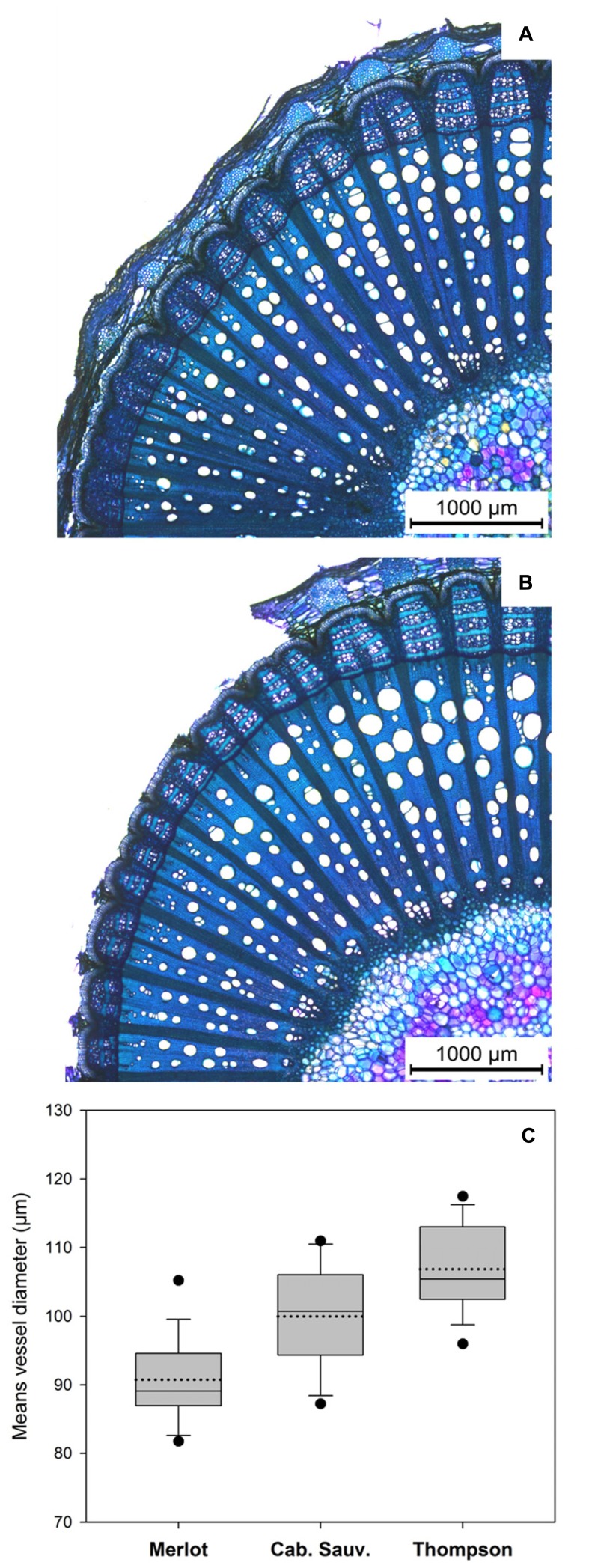
**Morphological and physiological features of the xylem of one year-old *Vitis vinifera* stems cvs. Merlot and Cabernet Sauvignon. (A,B)** Micrograph of cross-section of stem of *V. vinifera* cvs. Merlot **(A)** and Cabernet Sauvignon **(B)**. **(C)** Box plot representing means of equivalent circle diameters of LV measured in Merlot, Cabernet Sauvignon, and Thompson Seedless cultivars (*n* = 18): the median and mean are represented by solid and dotted lines, respectively. Top and bottom lines of the box correspond to the 25th and 75th percentiles of the data, respectively. Error bars represent the 10th and 90th percentiles. Circles represent outliers.

We hypothesized that in grapevine, the mechanism of tolerance toward esca disease is similar to that displayed by elms toward *O. ulmi* and *O. novo-ulmi*. We first looked at the wood anatomy in *V. vinifera* cvs. Merlot, Cabernet Sauvignon, and Thompson Seedless, and compared their morphological characteristics (see **Figures 4A,B**). These three cultivars were selected because many reports suggest they vary in tolerance to fungal vascular diseases, and represent a continuum between resistance and extreme susceptibility (**Table 1**). Merlot is relatively tolerant and Cabernet Sauvignon is relatively susceptible toward both esca and eutypa dieback (Péros and Berger, 1994; Christen et al., 2007; Rolshausen et al., 2008; Bruez et al., 2013; Lambert et al., 2013; Murolo and Romanazzi, 2014). Thompson Seedless, a white table grape cultivar, was shown to be more susceptible than Cabernet Sauvignon to esca disease and esca-associated pathogens, and can be considered as extremely susceptible (Feliciano et al., 2004; Travadon et al., 2013). Morphological analysis indicates that the mean of the diameter of LV in the stem of each cultivar differs significantly (**Figure 4C**; **Table 1**). Amongst many morphological traits we screened, including equivalent circle diameter of vessels, vessel density, and vessel grouping index, the mean vessel diameter is the only variable that can explain differences in cultivar tolerance. Merlot, the most tolerant cultivar, showed the lowest mean vessel diameter, whereas Thompson Seedless, the most susceptible, showed the greatest mean vessel diameter. Cabernet Sauvignon, the intermediate cultivar in term of susceptibility, showed an intermediate vessel diameter value. These findings are consistent with the relationship found in the DED pathosystem, whereby plant genotypes with small xylem vessels are more resistant to fungal wilt disease pathogens.

**Table 1:** Mean of equivalent vessel diameter measured in 1 years old stems of *V. vinifera* cvs. Merlot, Cabernet Sauvignon, and Thompson Seedless.

*Vitis vinifera* cvs.	Merlot	Cabernet Sauvignon	Thompson Seedless
Level of susceptibility to fungal vascular diseases	Low	Medium	High
Mean vessel diameter	90.7 ± 5.8 a	99.9 ± 7.1 b	106.9 ± 6.3 c

*Different letters indicate that significant differences (*P* < 0.01) were obtained using a Student’s test (*n* = 18).*

The advantage of small size vessels in plants is notable in the response to vascular infection. Many observations indicate that the chronic form of esca implies translocation of toxins from infected woody tissues to leaves (Bruno and Sparapano, 2007; Bruno et al., 2007; Andolfi et al., 2009; Luini et al., 2010). Restriction of fungi and toxin movement is assured with the plugging of infected vessels by tyloses and gels. Plant failure to develop tyloses and gels in infected conduits will increase their susceptibility toward vascular wilt pathogens (Yadeta and Thomma, 2013). The cylindrical volume of material necessary to fully occlude a known vessel length increases with square of the vessel diameter, such that a slight increase of the vessel diameter requires a substantial increase of the material synthesized to fully occlude the vessel. In grapevine, it has been reported that vessel occlusion following wounding is strongly reduced in the zone where conjunctive tissues react, suggesting that resource allocation could be subjected to a trade-off between vessel occlusion and defense reaction in conjunctive tissues (Pouzoulet et al., 2013). The need to synthesize a greater amount of occluding material might therefore lower the resources available to establish responses in conjunctive tissues at the vessel periphery. So, plants carrying vessels of small diameter like Merlot, might be able to restrict the spread of toxins and bud cells in a quicker and more efficient manner than plants carrying wider vessels like Cabernet Sauvignon and Thompson Seedless. In addition, small size vessel cultivars might be able to allocate their resources to compartmentalize fungi within conjunctive tissues more efficiently. As we discussed before, tyloses are subjected to a process of maturation. Incomplete or delayed maturation of tyloses will lead to less efficient compartmentalization of pathogens in vessels, and by consequence, enhance susceptibility to diseases.

Another possible scenario is that differences in xylem morphology affect disease expression through modifying xylem tolerance to drought stress. An important concept in xylem physiology is that a trade-off occurs between water transport efficiency, measured as maximum rates of hydraulic conductivity, and safety, measured as resistance to drought-induced xylem embolism (Hacke and Sperry, 2001; Wheeler et al., 2005; Hacke et al., 2006). Inter-specific studies indicate that resistance to drought-induced cavitation is correlated with xylem vessel diameter and length (Wheeler et al., 2005; Hacke et al., 2006; Sperry et al., 2006). Correlation between vessel dimension and xylem vulnerability to drought was also often observed at the intra-specific level (Sperry and Saliendra, 1994; Wheeler et al., 2005), and for the same genotype subjected to different abiotic condition (Plavcova et al., 2013). According to the air seeding theory, these are indirect relationships as resistance to cavitation is truly a function of the intervessel pit pores (Wheeler et al., 2005; Hacke et al., 2006). Larger vessels tend to have greater total pit area, which increases the probability that large pores occur in pits at the intervessel junction. Large pores allow cavitation at a lesser negative water potential, hence resulting in a greater vulnerability to embolism (Choat et al., 2008). The low wood density of grape vine implies that greater amounts of water can be transported through trans-sectional xylem area than the denser wood of trees. A high percentage of long and wide vessels is found in the xylem of grapevine (Wheeler et al., 2005), which helps to conduct large volumes of water throughout the plant. Vessels also present large scalariform perforations and often have a high pit area per vessel, which would suggest a greater vulnerability to embolism (Wheeler et al., 2005; Jansen et al., 2009; **Figure 2B**).

Grapevine xylem is an extreme case of efficiency/sacrificial strategy for water transport and is therefore particularly vulnerable to drought stress-induced xylem cavitation. Contrasting responses to water deficit and levels of vulnerably to embolism have also been described amongst cultivars of grapevine (Alsina et al., 2007). However, no correlation has been clearly established between vulnerability to drought-induced cavitation and susceptibility to fungal vascular wilt diseases. Such correlation was not found in DED, but we believe that it needs to be further investigated in grapevine and its susceptibility toward esca. The chronic form of esca includes tiger-stripe patterns on leaves and necrotic spots on berries, but is not a consequence of disruption of water transport (Bruno and Sparapano, 2007; Bruno et al., 2007; Andolfi et al., 2009). Moderate or short drought stress may induce a limited number of embolized vessels (Brodersen et al., 2013b). When drought conditions cease with a return to favorable hydric conditions, the refilling of these vessels occurs. The process of vessel refilling is not fully understood but might require an import of compounds having osmotic property, most probably sugars, in the vessel lumen in order to assure the entry of water from adjacent cells (Brodersen et al., 2010). This oxygen- and nutrient-rich environment might be conducive for pathogen growth and toxin production. Once water conductivity is reestablished, the systemic colonization of open vessels by fungal spores as well as the translocation of fungal toxins in the transpiration stream to the leaves could explain the development of observed tiger-stripe symptoms. Thus, cultivars with small xylem vessels such as Merlot, are less likely to express foliar symptoms because a limited drought-induced xylem cavitation will have occurred initially.

In addition, the acute form of the esca disease, which causes apoplexy in mature vines, or Petri disease, which causes apoplexy in young vines, do appear to be associated with drought stress and arrest of water transport (Edwards et al., 2007a; Spagnolo et al., 2011). Field observations indicate that apoplexy is favored following low rainfall, or can be associated with hot windy periods (Surico et al., 2000, 2006; Larignon et al., 2009). All of these conditions tend to increase evapo-transpiration, producing favorable conditions for the development of xylem embolism. It has been shown in the case of the Petri disease that fungal infection leads to substantial loss of trans-sectional area of sap conductive xylem. Moreover, the associated disruption of water transport widely extends to non-symptomatic areas of xylem, suggesting that embolism in non-infected vessels largely contributes to the global disruption (Edwards et al., 2007a). Under drought stress, stomatal conductance was shown to be higher and water potential lower in *P. chlamydospora* infected plants compared to non-infected ones (Edwards et al., 2007b,c). Such stomatal dysfunction takes place in other vascular wilt pathosystems as well (Tyree and Sperry, 1989) and appears to favor increased rates of water loss that potentially lead to xylem embolism. In this context, it can be hypothesized that plants that are less vulnerable to drought-induced embolism, like Merlot, will also respond to infection during drought stress more effectively and are less sensitive to apoplexy or wilt. Finally, increased susceptibility to drought could also predispose the plant to latent infection by opportunistic pathogens that thrive under plant stress conditions. Interaction between diseases and environmental or physiological stress cannot be excluded and should be further investigated.

## FACTORS INFLUENCING XYLEM VESSEL SIZE

Xylem formation is a highly complex process and is responsive to environmental changes. For instance, water availability during plant growth induces xylem acclimation with structural modification of vessels. This mechanism is likely to be conserved in woody plants. Indeed, several reports have shown that water availability induces modification of vessel diameter in *Ulmus* genotypes (Solla and Gil, 2002a), *Populus* genotypes (Fichot et al., 2009), *Malus domestica* (Bauerle et al., 2011) and *V. vinifera* (Lovisolo and Schubert, 1998). In addition, the degree of developmental plasticity of the xylem in response to water availability is a function of plant genetic makeup (Fichot et al., 2009). Such a structural modification of xylem vessels was shown to impact the expression of DED (Solla and Gil, 2002a). Acclimation to water availability should also affect the susceptibility to the disease in other pathosystems, such as esca disease in grapevine. One feature of chronic esca in natural vineyard settings is in the variability in symptom expression, whereby tiger-stripe symptoms on leaves and necrotic spots on berries show up one year but disappear in another. It has been shown that chronic esca incidence in the field is correlated with the amount of annual precipitation occurring in the spring, when vines achieve vegetative development and perennial structure formation (Marchi et al., 2006; Calzarano et al., 2009; Guérin Dubrana et al., 2013). We can suspect that these annual changes in symptom expression are due to differences in the size of new vessels formed under different water regimes and vigor conditions. Interestingly, other abiotic factors promoting plant growth, such as fertilization, were reported to have a positive impact on esca disease expression (Calzarano et al., 2009).

*Vitis vinifera* commercial wine grapes (a.k.a., scions) are commonly grafted onto rootstocks. Grapevine rootstocks are individual *Vitis* species or most frequently crosses of two or more species that are not of *V. vinifera* parentage. Rootstocks provide many benefits, such as drought stress tolerance and resistance to diseases and pests, but also impact plant vigor and nutrient assimilation (Koundouras et al., 2008; Keller, 2010; Gambetta et al., 2012). Correlation between vessel diameter, maximal hydraulic conductivity in roots and trunks of rootstocks and their ability to confer vigor to scions were observed in many perennial crops such as *Prunus* spp. (Gonçalves et al., 2007; Tombesi et al., 2010b; Tombesi et al., 2011), *Malus* spp., (Bauerle et al., 2011), and *Olea* spp. (Trifilò et al., 2007). However, rootstock-mediated vigor enhancement or reduction does not necessarily come with a change in vessel diameter in the scion (Tombesi et al., 2010a; Bauerle et al., 2011). Recently, Murolo and Romanazzi (2014) proposed that significant variation in esca disease expression could be attributed to vine rootstocks. These authors attributed differences in observed disease expression to variation in drought stress tolerance of rootstocks tested. In grapevine, the control of scion water status by rootstocks in conditions of water deficit is assured through two main physiological processes related to the regulation of stomatal closure and root hydraulic traits (Tramontini et al., 2013). The regulation of transpiration-mediated stomatal closure is under control of absissic acid (ABA), secreted at the root level in response to drought deficit (Lovisolo et al., 2010). Marguerit et al. (2012) showed that the transpiration rate and acclimation to water deficit conferred by rootstocks to scions were not related and were under the control of different genes in rootstocks. At the hydraulic level in roots, radial water movement mediated by aquaporins was shown to have a significant potential contribution in drought stress adaption (Lovisolo et al., 2008, 2010; Vandeleur et al., 2009), but also affects root hydraulic conductance (Perrone et al., 2012), water uptake by fine roots (Gambetta et al., 2012) and by consequence plant growth.

## PERSPECTIVES

We suggest that an integrated vision of the role of xylem biophysical properties in resistance to abiotic and biotic stresses is important for understanding or controlling how these factors interact in a variety of plant systems. For example, the implementation of a sustainable management program for esca disease that combines planting of grapevine cultivars with small vessels and appropriate water regimes as well as controlled plant vigor could mitigate some of the economic losses encountered by the grape industry. In addition to *O. ulmi* and *O. novo-ulmi*, fungal wilt pathogens such as *Fusarium* spp. And *Verticillium* spp. are the cause of many economically important diseases in annual and perennial crops, ornamental plants and forest trees. If the correlation between vessel size and disease susceptibility is consistent across different pathosystems, then disease management could be achieved by using the strategies proposed above.

Beyond grapevine, diseases and water deficit interact to limit productivity or induce mortality across a wide range of plant systems. For example, recent mortality of woody species during global change-type droughts involves individuals weakened by drought stress increasing in susceptibility to attack by pests and pathogens (McDowell et al., 2008; Allen et al., 2010). Thus inter-disciplinary approaches between grapevine pathology and tree eco-physiology have the potential to provide a better understanding of environmental and agricultural issues, and can potentially open the path to alternative cultural practices and disease management strategies. In the actual context of climate change, deciphering how perennial plants adapt to drought is of crucial concern for future crop water management and improved water use efficiency. In this scheme, the role of xylem plasticity within individual plants and among plant species and its consequences for xylem vulnerability to drought stress and susceptibility to vascular pathogens, offer enticing avenues for better understanding biotic and abiotic resistance at the genetic, cellular and whole plant levels of organization.

## MATERIALS AND METHODS

One year-oldstems of *V. vinifera* cvs. Merlot [Foundation Plants Service (FPS) selection 06], Cabernet Sauvignon (FPS selection 31) and Thompson Seedless (FPS selection 02A) were provided by the FPS (University of California, Davis, USA; http://fps.ucdavis.edu/). Mother-plants sampled were own rooted, and were 17–21 years old, 18 years old, and 15 years old; for Merlot, Cabernet Sauvignon, and Thompson Seedless, respectively. Stems were sampled in March of 2013 and 2014. In 2013, 12 stems (3 stems coming from 4 different mother-vines) were analyzed for each cultivars. In 2014, the experiment was repeated with 6 stems per cultivar (3 stems coming from 2 different mother-vines). Internodes ranging from 8 to 10 mm in diameter, and 100 to 120mm in length were selected. A stem segment of about 10 mm in length was sampled in the middle part of each internodes, and fixed in ethanol 80%. Cross-sections of 70 μm were obtained as described by Pouzoulet et al. (2013). Section were stained with toluidine O as described by Ruzin (1999) and observed using a bright field microscope (DM4000, Leica Microsystems CMS GmbH, Wetzlar, Germany). Micrographs (100X magnification) were assembled using LAS v4.2 (Leica Microsystems CMS GmbH, Wetzlar, Germany) in order to create high definition pictures covering a quarter of each stem section. Morphological measurement were realized using LAS v4.2. For each stem, large vessel areas were determined for three FP 45° from each other, so that one fascicular portion analyzed was positioned on the dorso-ventral symmetry axis, and another one on the lateral symmetry axis. Data collected from FP were pooled by stems. Vessel areas were converted to their arithmetic diameters according to Scholz et al. (2013) and mean vessel diameters of each stem were determined. Data collected from 2013 and 2014 stems (18 stems per cultivars) were pooled and mean vessel diameters and standard deviations of each cultivar were determined. Statistical analysis were carry out through a Student test using EXCEL 2007 (Microsoft Corporation, Redmond, WA, USA).

## Conflict of Interest Statement

The authors declare that the research was conducted in the absence of any commercial or financial relationships that could be construed as a potential conflict of interest.
